# A meta-analytic evaluation of the reliability of work-family and family-work conflict scales

**DOI:** 10.1038/s41598-024-83086-z

**Published:** 2024-12-30

**Authors:** Lawrence Ejike Ugwu, Erhabor Sunday Idemudia

**Affiliations:** Faculty of Humanities, North-West University, Potchefstroom, South Africa

**Keywords:** Work-Family Conflict, Family-Work Conflict, Reliability Generalization, Meta-Analysis, Conservation of Resources Theory, Psychometric Properties, Psychology, Human behaviour

## Abstract

In occupational psychology, measuring the interplay between work and family roles is crucial. The Work-family conflict (WFC) and Family-Work Conflict (FWC) scales are indispensable tools in this endeavour. However, their reliability across diverse geographical locations and work settings, particularly during the global pandemic, requires thorough examination. This meta-analysis evaluates the reliability generalisation of WFC and FWC scales across different studies. Registered with PROSPERO (ID: CRD42024509030), we systematically searched four major databases: EBSCOhost, Web of Science, Scopus, and ScienceDirect, yielding 506 potential studies. After deduplication and screening, 44 studies published between 2000 and 2024 were selected. These studies analysed reliability coefficients (Cronbach’s alpha) and the impact of geographical location, work setting, and events like the COVID-19 pandemic on scale reliability. The analysis showed high reliability for both scales, with a pooled Cronbach’s alpha of 0.91 (95% CI [0.87, 0.95]) indicating strong internal consistency. Test-retest reliability was also high, with a pooled estimate of 0.89 (95% CI: [0.84, 0.93]). Subgroup analyses revealed minimal variability in reliability across sectors, regions, and the pandemic period, with no heterogeneity (I^2^ = 0.0%), suggesting the scales’ broad applicability. The WFC and FWC scales demonstrate high reliability across various global contexts, underscoring their value in occupational psychology. Their adaptability to different cultural and work scenarios, including during the COVID-19 pandemic, highlights their potential for widespread use. The uniform reliability across studies emphasises their effectiveness in capturing work-family dynamics, supporting their generalizability and relevance for cultural adaptation.

**Supplementary Information:**

The online version contains supplementary material available at 10.1038/s41598-024-83086-z.

## Introduction

The relationship between work and family life has garnered significant scholarly attention due to its profound effects on employee well-being and organisational performance. This interest is primarily because it is universally recognised that work-family dynamics significantly affect individuals’ quality of life and organisation outcomes^1^. Work-Family Conflict (WFC) and Family-Work Conflict (FWC) are crucial in exploring individuals’ challenges in juggling their professional and personal responsibilities^2^. Specifically, WFC occurs when work obligations encroach upon family life, whereas FWC describes situations where family duties negatively impact work performance^3^.

The development and validation of scales measuring WFC and FWC have significantly advanced our understanding of these conflicts. These tools emphasise the multifaceted nature of work-family dynamics and have illuminated how work and family life intersect, affecting individual stress levels, job satisfaction, and family relationships^1,4^.

Reliability Generalisation (RG), a meta-analytic technique, plays a crucial role in evaluating the consistency of these measurement instruments across studies. RG assesses the distribution of reliability coefficients reported in the literature, identifying the factors that may influence these values, such as sample characteristics and measurement contexts^5,6^. This approach provides insights into the variability of scale reliability, thereby enabling researchers and practitioners to gauge the robustness and applicability of these instruments in various settings and among different populations.

Research has extensively documented the reliability of WFC and FWC scales, affirming their psychometric soundness across diverse samples. Notable studies have reported satisfactory Cronbach’s alpha values, indicating strong internal consistency, and have validated the scales against related constructs, thus supporting their criterion and construct validity^7^.

In corporate and organisationals contexts, WFC and FWC scales have been extensively used to explore the impact of job demands, work schedules, and organisational culture on employees’ work-family balance^3^. These studies highlight the efficacy of flexible working arrangements and supportive organisational policies in mitigating conflict and enhancing employee satisfaction and productivity^8^.

The healthcare sector, known for its demanding work environments, has been a pivotal setting for investigating work-family conflicts. Studies involving nurses, doctors, and other healthcare professionals have uncovered the significant impact of shift work, emotional labour, and job stress on WFC and FWC, with implications for job satisfaction, burnout, and patient care quality^9^.

Cross-cultural research using WFC and FWC scales has offered valuable insights into how cultural norms and values shape the experience and reporting of work-family conflicts. Such studies have unveiled differences in the prevalence and predictors of WFC and FWC across countries, reflecting variations in societal expectations, family roles, and support systems^10^.

With the rise of telecommuting and remote work arrangements, recent research has focused on their implications for work-family conflicts. These studies examine how the blurring of work and family boundaries in remote work settings affects the applicability and interpretation of WFC and FWC scales^11^.

In educational settings, using WFC and FWC scales among faculty and administrative staff has highlighted the unique challenges of balancing academic responsibilities with family life^12^. Despite the proliferation of research utilising these scales, variability in their reported reliability across studies, attributed to differences in sample characteristics, cultural contexts, and methodological approaches, underscores the necessity for a meta-analytic review^13^.

Therefore, this meta-analysis aims to aggregate reliability coefficients from existing studies on WFC and FWC scales, comprehensively evaluating their psychometric robustness. By identifying potential moderators of reliability, this study seeks to illuminate the conditions under which these scales demonstrate optimal reliability, thereby contributing to their refined application in research and practice. This effort addresses a critical gap in the literature, facilitating more complex assessments of work-family dynamics and informing interventions designed to mitigate conflict in these essential life domains.

Several theoretical perspectives provide valuable insights into understanding the mechanisms driving Work-Family Conflict (WFC) and Family-Work Conflict (FWC). The Conservation of Resources (COR) Theory, proposed by Hobfoll^14^, offers a compelling lens through which to view these conflicts. According to COR Theory, individuals strive to acquire and conserve valuable resources such as time and energy. WFC and FWC arise when these resources are threatened or depleted, leading to stress. This theory’s broad applicability may explain the high reliability of the WFC and FWC scales across diverse contexts, as these scales effectively measure the conflicts stemming from resource depletion^15^.

Role Theory also provides an essential framework for examining WFC and FWC. Initially developed by Kahn et al.^16^, It suggests that managing multiple roles with conflicting demands leads to strain. WFC occurs when work obligations interfere with family roles, while FWC arises when family responsibilities impede work performance. Greenhaus and Beutell^17^ noted that the consistent reliability of these scales suggests their effectiveness in capturing these widespread role conflicts.

Boundary Theory offers further insights, particularly in the context of the COVID-19 pandemic. As individuals increasingly blur the boundaries between work and family life, especially with the rise of remote work, conflicts intensify^18^. The WFC and FWC scales’ adaptability during the pandemic highlights their ability to capture these evolving dynamics, supporting the relevance of Boundary Theory^19^.

Additionally, Social Exchange Theory posits that WFC and FWC emerge when the perceived costs of participating in work and family roles outweigh the rewards^20^. The high reliability of the scales reflects their capacity to capture these imbalances, which are central to social exchange processes^21^.

Finally, the Job Demands-Resources (JD-R) Model explains how high job demands coupled with limited resources lead to stress and conflicts between work and family roles^22^. The model’s relevance across various sectors aligns with this study’s findings, as the WFC and FWC scales effectively measure conflicts arising from these universal stressors^23^.

## Objective

Ultimately, this study aims to (1) assess the overall reliability estimates of the WFC and FWC scales and (2) evaluate them across geographic and global crises and settings.

### Methods

We conducted a reliability Generalization Meta-Analysis (RG) to assess the psychometric properties of the work-family and family-work conflict scales. The current meta-analysis was pre-registered in PROSPERO. This meta-analysis included data from 44 studies, and the review method adhered to reliable COnsensus-based Standards for selecting health Measurement Instruments Risk of Bias checklist (COSMIN RB^24^).

#### Search strategy

The systematic review followed a predefined search strategy. Databases like EBSCOhost, Business Source Complete, Scopus, Web of Science, ScienceDirect and Medline were queried, and relevant studies published between 2000 and 2024 were included. Identify Keywords such as Reliability, WFC/FWC scales, Work-family and family-work scales, Measurement Properties, Assessment, Psychometric properties, Internal consistency, Cronbach’s alpha, and reliability (see S1 Table).

#### Selection criteria

The study aims to inclusively consider research on the psychometric properties and reliability assessment of the Work-family and family-work scales (WFC/FWC scales) across varied populations and settings, ensuring a comprehensive and representative sample. This inclusivity extends to inter-rater reliability, test-retest reliability, internal consistency, and across cultures. Given the global application of the WFC/FWC scales, the selection criteria are designed to promote diversity by encompassing studies that explore resilience in different demographic groups, cultural contexts, and geographic locations.

Exclusion criteria prioritise the relevance and reliability of the data, excluding studies that do not report on reliability or psychometric properties, publications not in English, reviews, conference abstracts, editorials, case reports, and those lacking sufficient data. This approach ensures a complex understanding of resilience across diverse contexts, contributing to a more representative and robust meta-analysis.

#### Data extraction

The selection of studies for Reliability Generalization (RG) adhered to specific eligibility criteria, involving two independent reviewers who screened titles, authors, publication details, Digital Object Identifiers (DOIs) or URLs, and abstracts of each study. Following this initial screening, full-text articles meeting the criteria underwent a comprehensive assessment for final inclusion, with any discrepancies resolved through consultation with an independent reviewer. The subsequent data extraction process focused on the psychometric properties of the Work-family and family-work scales (WFC/FWC scales). This encompassed gathering information on study characteristics, demographics, reliability coefficients, inter-rater reliability, and test-retest measures. While McDonald’s omega is considered a more robust measure of internal consistency, particularly in cases where tau-equivalence is not met, most studies included in this meta-analysis did not report McDonald’s omega. As such, we focused primarily on Cronbach’s alpha, the most consistently reported reliability metric across studies. Where possible, future research should prioritise reporting both Cronbach’s alpha and McDonald’s omega to provide a more comprehensive reliability assessment. Extracting data on sample characteristics, including size and participant demographics, enabled a subtle interpretation of reliability across diverse populations and contributed to the overall synthesis of findings (see Supplementary File 1 for more information). The extraction process also considered potential moderators, including language version, age, gender, and cultural factors. The entire process, from study selection to data extraction, was meticulously documented for consistency and accuracy. In disagreements, consensus was reached through collaborative discussions between reviewers, ensuring a robust and reliable foundation for the meta-analysis.

#### Quality assessment

Two independent investigators conducted using Quality Assessment of Diagnostic Accuracy Studies (QUADAS-2^25^), and COnsensus-based Standards for the selection of health Measurement Instruments Risk of Bias checklist (COSMIN^24^), to assess the quality of the included studies. We conducted a comprehensive evaluation of multiple studies using the QUADAS-2 framework, providing critical insights into the methodological quality of each study (S1. Fig). The categories, including patients’ selection, Index Text, Reference Standard, Flow and Timing, and Overall Assessment, offer insight into each study’s methodological rigour and potential biases. QUADAS-2 tool was used to rate each included survey on a 4-point scale: “Low”, “Some concern”, and “High”.

The Quality Assessment of Diagnostic Accuracy Studies (QUADAS-2^25^), and the COnsensus-based Standards for the selection of health Measurement Instruments Risk of Bias checklist (COSMIN RB^25^), (S2 Table). The QUADAS-2 framework, employed for diagnostic accuracy studies, allows for a detailed analysis of patients’ selection, Index Test, Reference Standard, Flow and Timing, and Overall Assessment, shedding light on methodological rigour and potential biases in each study. Utilising the QUADAS-2 tool, each included study underwent a rigorous evaluation on a 4-point scale, categorising the assessments as “Low,” “Some concern,” or “High.” Notably, the studies under consideration received a “Low” classification, indicating a favourable outcome regarding methodological quality, reinforcing their credibility and reliability in the context of the assessment criteria^25^.

To assess the included studies using the COSMIN Risk of Bias (RB) checklist, attention is directed toward crucial aspects of the questionnaire’s measurement properties. As outlined by Mokkink in 2018, COSMIN RB primarily scrutinises reliability and responsiveness. The COSMIN RB checklist comprises ten checkboxes, each corresponding to specific metric properties. Each checkbox contains items addressing various aspects of design and statistical methods.

The studies included in the evaluation were systematically rated for reliability and responsiveness (S1 Table). The classifications for these assessments are denoted as “Very Good,” “Adequate,” or “Doubtful.” These ratings provide a detailed understanding of each study’s methodological strengths and potential limitations, contributing valuable insights into the reliability and responsiveness of the measurement properties under investigation. Methodological quality significantly impacts meta-analysis findings. The robust methodologies of each included study contribute to the reliability and precise estimates, enhancing the overall credibility and generalizability of the meta-analysis on WFC/FWC scales in this study.

### Data analysis

In data analysis, we employed meta-analysis statistical methods to synthesise findings across studies. Considering heterogeneity, effect sizes for reliability coefficients were calculated using random-effects models. A qualitative analysis will summarise the reliability of the work-family and family-work scales. Additionally, a meta-analysis employing a random-effects model will pool reliability coefficients. Heterogeneity was explored through the I^2^ statistic and subgroup analyses. Data analysis was conducted using R Studio with the metafor package. This meta-analysis calculated effect sizes based on Cronbach’s alpha, which measures a scale’s internal consistency or reliability.

This approach allows for synthesising findings regarding the reliability of WFC/FWC scales across multiple studies. A random-effects model was chosen due to the likelihood of heterogeneity among studies. The random-effects model accounts for variations in true effects across different populations or contexts. This choice acknowledges that the included studies may have different underlying true effects, making the random-effects model more suitable for generalising findings beyond the specific studies included. Heterogeneity among studies was assessed using the I^2^ statistic. I^2^ quantifies the percentage of total variation across studies due to heterogeneity rather than chance. Higher values of I^2^ indicate greater heterogeneity. The random-effects model accounts for variability between studies, providing a realistic estimate of the average effect size across different contexts^26^. Critics argue it may overestimate heterogeneity^27^, but when heterogeneity is minimal, random-effects and fixed-effects models yield similar estimates, reinforcing our findings’ robustness^28^.

Subgroup analyses were conducted to explore potential sources of heterogeneity, such as variations in study design, participant characteristics, or other factors. Addressing heterogeneity is crucial as it impacts the interpretation and generalizability of the meta-analysis results.

Egger’s test was used to assess funnel plot asymmetry, which may suggest publication bias due to small-study effects. The test involves regressing the standard normal deviation of the effect size on its standard error, and a significant intercept suggests asymmetry, indicative of bias. Additionally, the trim-and-fill method was employed to estimate the number of missing studies and to adjust the overall pooled effect size, accordingly, providing a conservative estimate of the effect size in the presence of publication bias.

High heterogeneity suggests variability in reliability estimates among studies. It prompts a careful interpretation of the overall findings, recognising that the true reliability of WFC/FWC scales may differ across diverse populations or under various conditions. Subgroup analyses help identify factors contributing to heterogeneity, offering insights into the sources of variation. R Studio, a comprehensive, integrated development environment, was utilised for its flexibility and powerful tools. The metafor package in R Studio facilitated the implementation of the meta-analysis—specific functions such as ‘**metagen()’** combined effect sizes and estimated overall effects. The ‘**forest ()’** function created the forest plot, visually representing the meta-analysis results. The code structure ensures transparency and allows for further customisation or adaptation based on the specific needs of the analysis.

### Ethical considerations

Given the unique nature of our study, we did not secure formal ethical approval before its commencement. However, in our meticulous effort to assess the reliability generalisation of the WFC/FWC scales via meta-analysis, we remained steadfast in our commitment to ethical principles. This commitment entailed safeguarding the confidentiality of participant data, securing informed consent, and upholding the integrity of our research. Additionally, our dedication to maintaining transparency and methodological rigour is demonstrated through our compliance with the PROSPERO protocol registration and applying PRISMA guidelines (PRISMA Checklist see S3 table). These measures ensure that our research approach is comprehensive and responsible, fostering trust within the scientific community about the credibility and dependability of our results.

The 44 studies finally screened for the meta-analysis included data from diverse geographical locations covering the healthcare, education, hospitality, and sales sectors. Sample sizes ranged from 20 to 1416 participants, highlighting the study’s broad scope.

Cronbach’s alpha scores for WFC and FWC suggest robust internal consistency across studies, with scores predominantly above 0.80. The studies reflects a mix of population characteristics, with certain studies focusing on specific professional groups, thereby providing sector-specific insights into work-family dynamics.

## Results

### Reliability report of the included studies

**Fig. 1:**
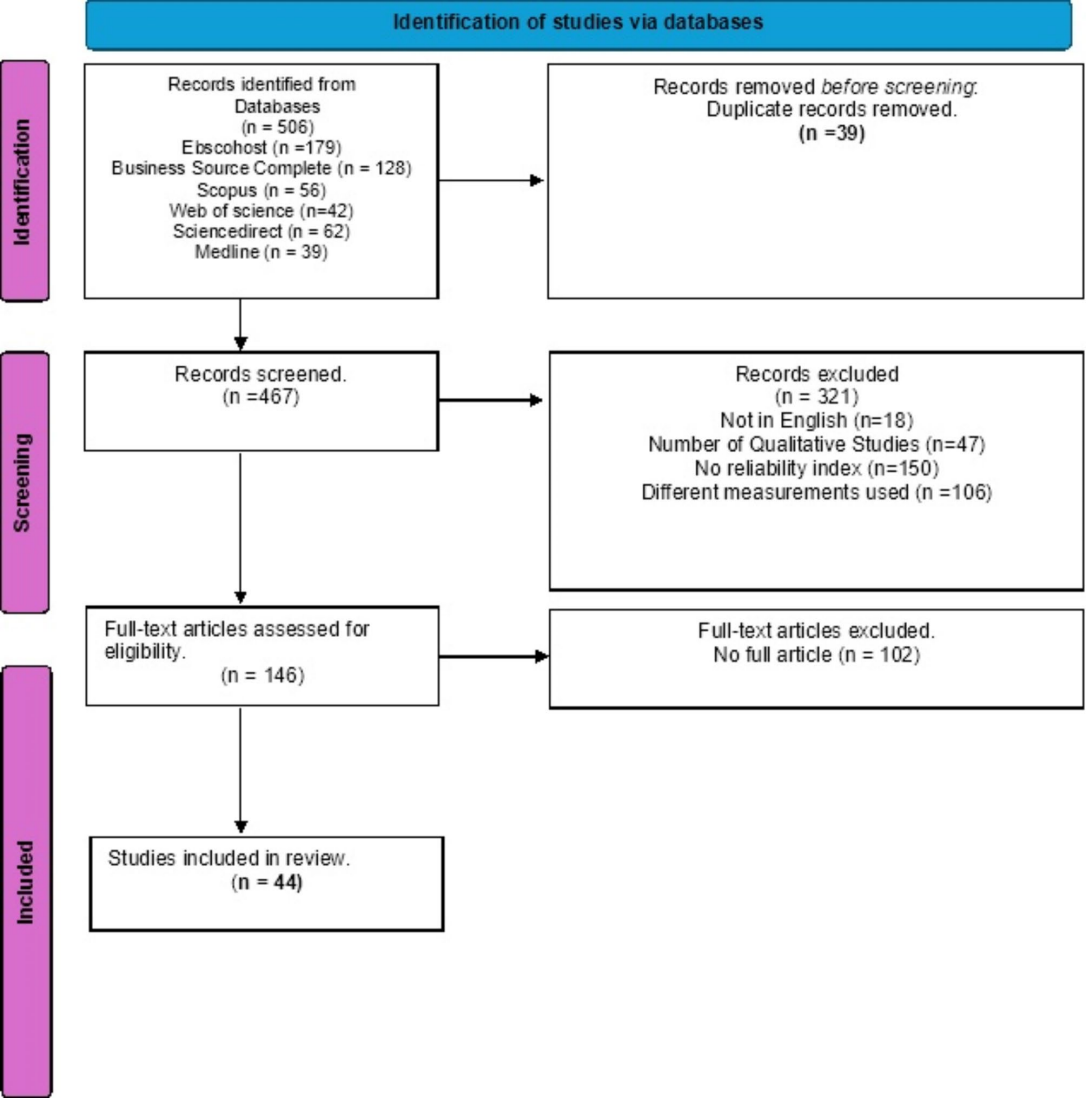
PRISMA diagram of the search and selection process.

A total of 506 research articles were identified by searching databases (see Fig. 1). These identified 506 research articles in the database, 321 (63.44%) of the identified articles were discarded based on the study exclusion criteria, 39(7.08%) studies were duplicated, and 146(28.85%) full articles were assessed for eligibility. However, 44(8.70%) studies were finally included in the meta-analysis based on the study inclusion criteria. The 44 studies in the reliability generalisation (RG) meta-analysis reported that the reliability coefficient (Cronbach’s Alpha) ranged from 0.66 to 0.96 for WFC/FWC.

### Reliability generalisation meta-analysis

**Table 1:** Summary of random-effects model of WFC/FWC reliability generalisation meta-analysis.

Total scales	k	Estimate α_+_	zval	95%CL	Q	I^2^
				LL(UL)		
Coefficient alpha						
WFC						
Common Model	44	0.91	45.75	0.87(0.95) ***	6.89	0.0%
Random Model		0.91	45.75	0.87(0.95) ***		
FWC						
Common Model	44	0.91	39.57	0.85(0.93)***	8.33	0.0%
Random Model			39.57	0.85(0.93)***		

Note: k = number of studies, Q = Cochran’s Heterogeneity Q statistics, I^2 =^ Heterogeneity index, *** *p* < 0.001.

In our meta-analysis exploring the reliability of Work-Family Conflict (WFC) and Family-Work Conflict (FWC) as subscales (see Table 1) within a broader scale aimed at understanding the interplay between work responsibilities and family life, we scrutinised data across 44 studies for each subscale. This analysis was critical in assessing the scales’ consistency in capturing the essence of conflicts between work and family spheres.

**Fig. 2:**
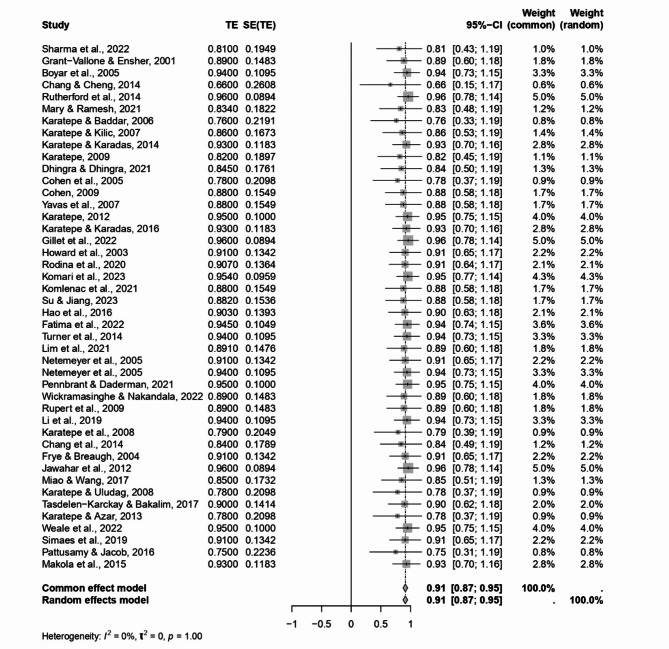
Forest plot concerning the WFC dimension Cronbach’s alphas.

For the WFC subscale, the findings indicated a high level of internal consistency, with a weighted mean Cronbach’s alpha (α+) and a weighted mean (ωα+) both calculated at 0.91. The robustness of this estimate is further supported by a significant z-value of 45.75 and a 95% confidence interval (CI) stretching from 0.87 to 0.95 (see Table 2; Fig. 2). This high internal consistency underlines the WFC subscale’s reliability in assessing conflicts stemming from work responsibilities encroaching on family life.

**Fig. 3:**
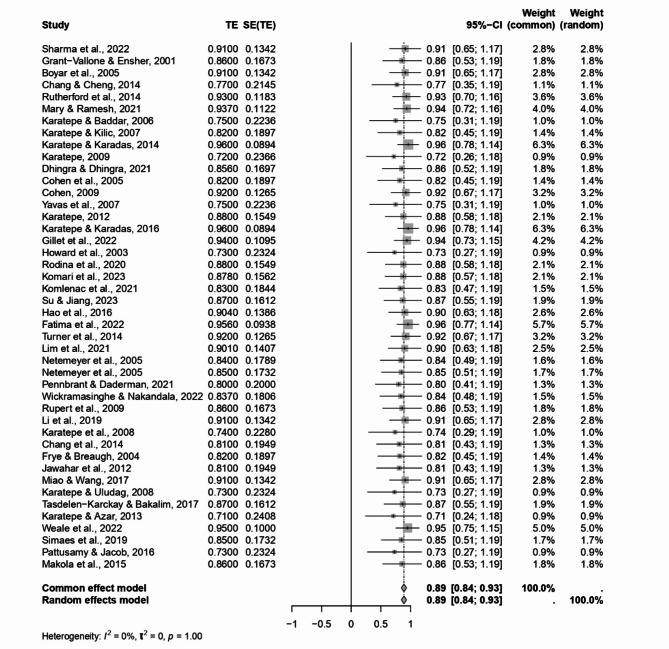
Forest plot concerning the FWC dimension Cronbach’s alphas.

Similarly, the FWC subscale demonstrated substantial internal consistency. The weighted mean Cronbach’s alpha (α+) for this subscale was also noted at 0.91, with the weighted mean (ωα+) slightly lower at 0.86. This consistency is affirmed by a significant z-value of 39.57 and a 95% CI ranging from 0.85 to 0.93. These statistics highlight the FWC subscale’s effectiveness in capturing the inverse conflicts, where family responsibilities impact work-related tasks (see Fig. 3).

The Cochran’s Heterogeneity Q statistics values (6.89 for WFC and 8.33 for FWC) were not significant, and the heterogeneity index (I^2^) for both subscales was observed at 0.0%. This suggests no heterogeneity in how work-family and family-work conflicts are measured across the studies included in our analysis.

These findings are crucial in exploring work-life balance, offering robust and reliable tools for assessing the bidirectional conflicts between work and family life. The significant Cronbach’s alpha values, coupled with the absence of observable heterogeneity (I^2^ index at 0.0%), reinforce the generalizability and applicability of the WFC and FWC subscales across different research settings. The 0.0% I^2^ values point towards consistency in measuring work-family and family-work conflicts, affirming these subscales’ utility in various research contexts focused on understanding and mitigating the challenges of balancing work and family responsibilities.

**Fig. 4:**
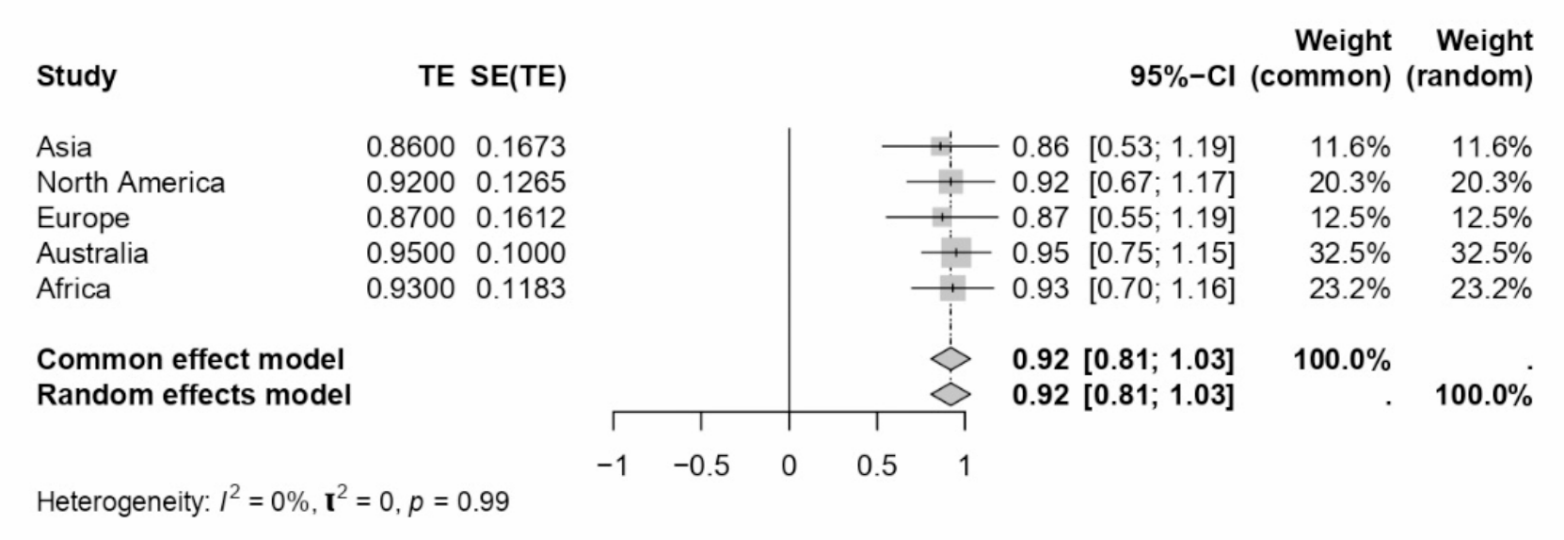
Forest plot concerning location-related WFC dimension Cronbach’s alphas.

The funnel plot for the meta-analysis of Cronbach’s Alpha values of the WFC and FWC scales (see Fig. 4) indicated no substantial evidence of publication bias, as the distribution of studies appeared symmetrical around the mean effect size. Larger studies with smaller standard errors clustered at the top of the plot, while smaller studies showed greater variability, forming an appropriate inverted funnel shape. Egger’s test for funnel plot asymmetry yielded a non-significant result (z = -0.09, *p* = 0.93), indicating no evidence of publication bias. The intercept estimate (b = 1.09, 95% CI: 0.85 to 1.33) further confirmed the lack of small-study effects. This suggests that smaller studies did not report systematically different reliability estimates compared to larger studies, reducing the likelihood of bias affecting our findings.

Similarly, the trim-and-fill method estimated that no studies were missing (SE = 3.92), and the adjusted pooled Cronbach’s alpha remained unchanged at 0.90, further supporting the robustness of the meta-analytic results. This suggests that the findings are unlikely to be substantially affected by publication bias, indicating that the meta-analysis provides a balanced representation of the available literature.

**Fig. 5:**
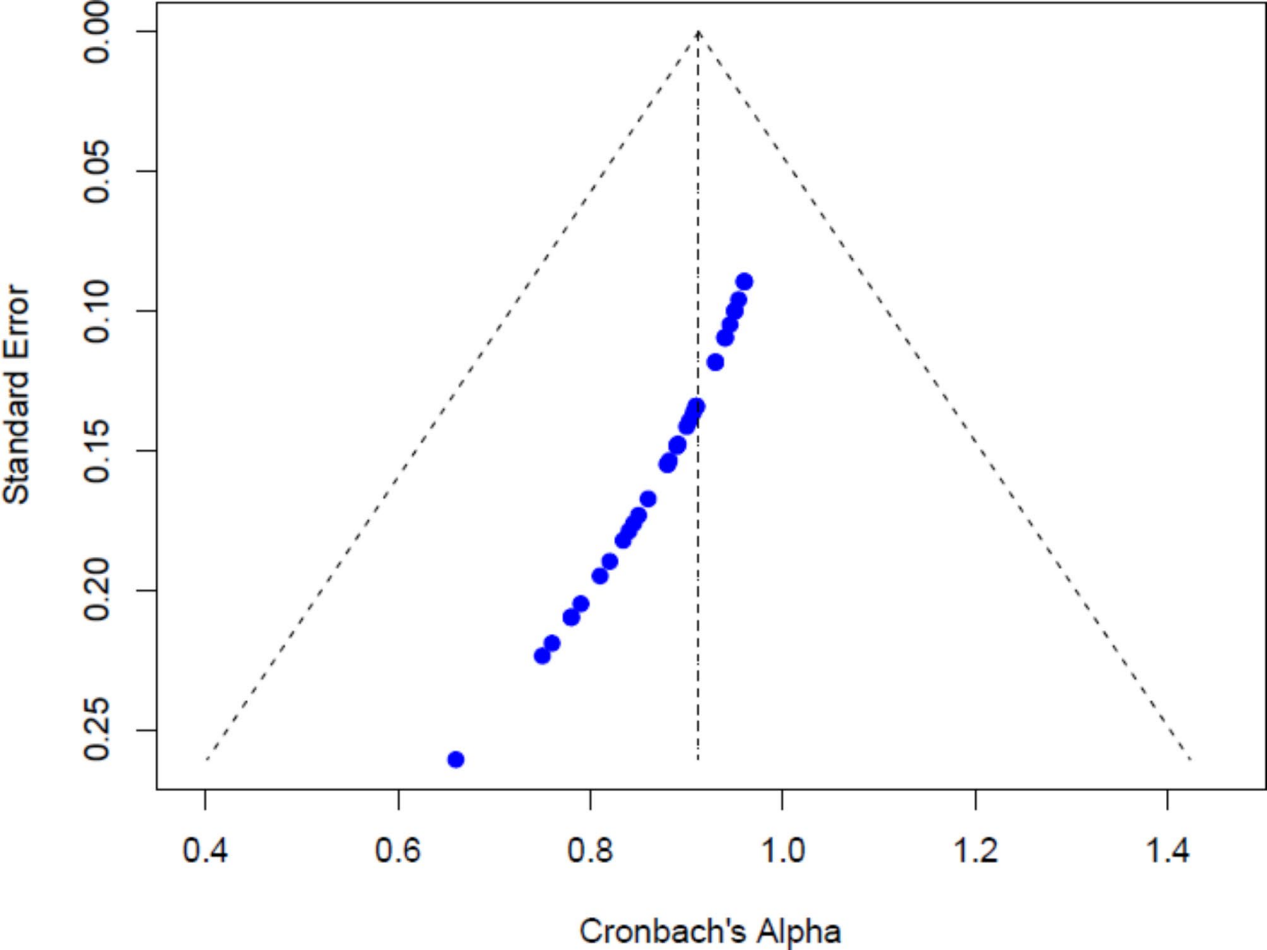
Funnel Plot for the WFC dimension.

The funnel plot (see Fig. 5) indicates a slight asymmetry, with studies clustering towards higher Cronbach’s Alpha values, suggesting that most report high WFC scale reliability. The skew toward higher reliability estimates may hint at a mild publication bias, where studies with lower reliability are underrepresented. However, the overall pattern still supports the consistency and high reliability across the included studies.

**Fig. 6:**
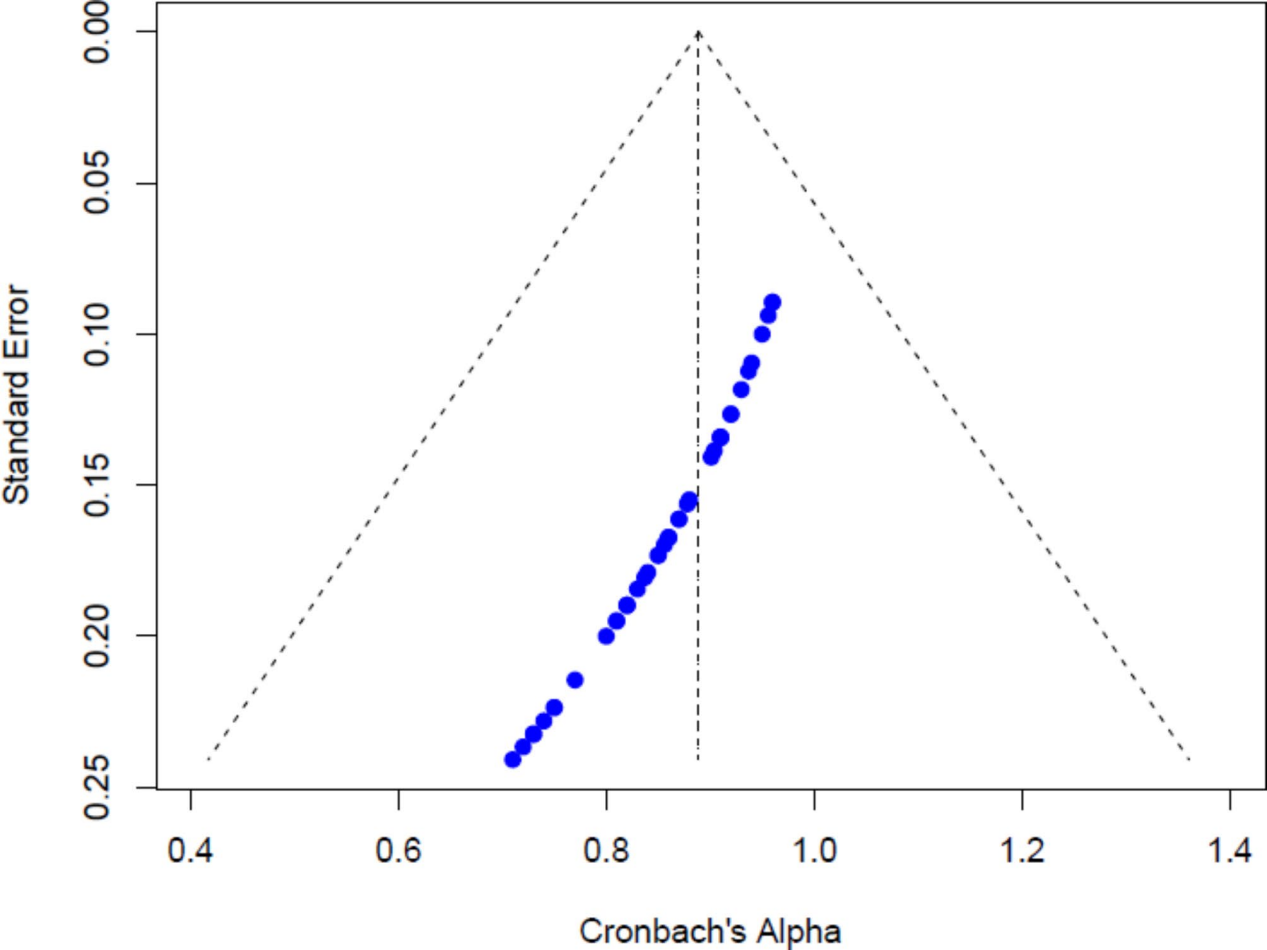
Funnel plot for FWC dimension.

For the FWC dimension, the funnel plot above (see Fig. 6) shows a pattern of slight asymmetry, with studies concentrated near higher Cronbach’s Alpha values, close to 1.0. This suggests that most studies report high reliability for the FWC scale. The asymmetry indicates a potential mild publication bias, where studies with lower reliability may be underrepresented. Nonetheless, the overall distribution still supports the consistency and reliability of the studies analysed.

**Table 2:** Subgroup analysis.

	K	WFC Estimate (95%CI)	FWC Estimate (95%CI)
SECTOR			
Health	12	0.91 [0.65; 1.17]	0.88 [0.58; 1.18]
Education	6	0.80 [0.41; 1.19]	0.84 [0.49; 1.19]
Hospitality	10	0.85 [0.51; 1.19]	0.80 [0.41; 1.19]
Customer service	2	0.93 [0.69; 1.17]	0.85 [0.50; 1.19]
Sales	2	0.91 [0.64; 1.18]	0.92 [0.67; 1.17]
Others	12	0.91 [0.65; 1.17]	0.86 [0.54; 1.19]
LOCATION			
Asia	13	0.86 [0.53; 1.19]	0.87 [0.55; 1.19]
North America	10	0.92 [0.67; 1.17]	0.86 [0.53; 1.19]
Europe	18	0.87 [0.55; 1.19]	0.83 [0.47; 1.19]
Australia	1	0.95 [0.75; 1.15]	0.95 [0.75; 1.15]
Africa	1	0.93 [0.70; 1.16]	0.86 [0.53; 1.19]
COVID			
PRE-COVID	31	0.870 [0.55; 1.19]	0.84 [0.49; 1.19]
COVID	1	0.91 [0.64; 1.17]	0.88 [0.58; 1.18]
POST-COVID	12	0.90 [0.62; 1.18]	0.89 [0.60; 1.18]

**Fig. 7:**
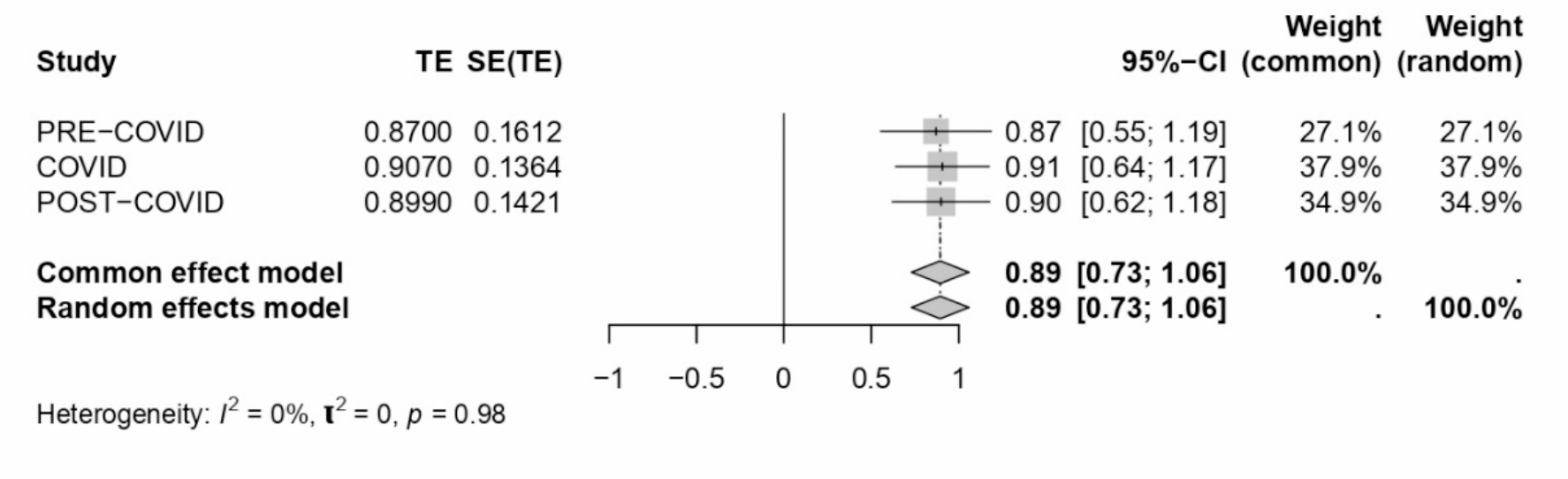
Forest plot concerning the COVID period WFC dimension Cronbach’s alphas.

**Fig. 8:**
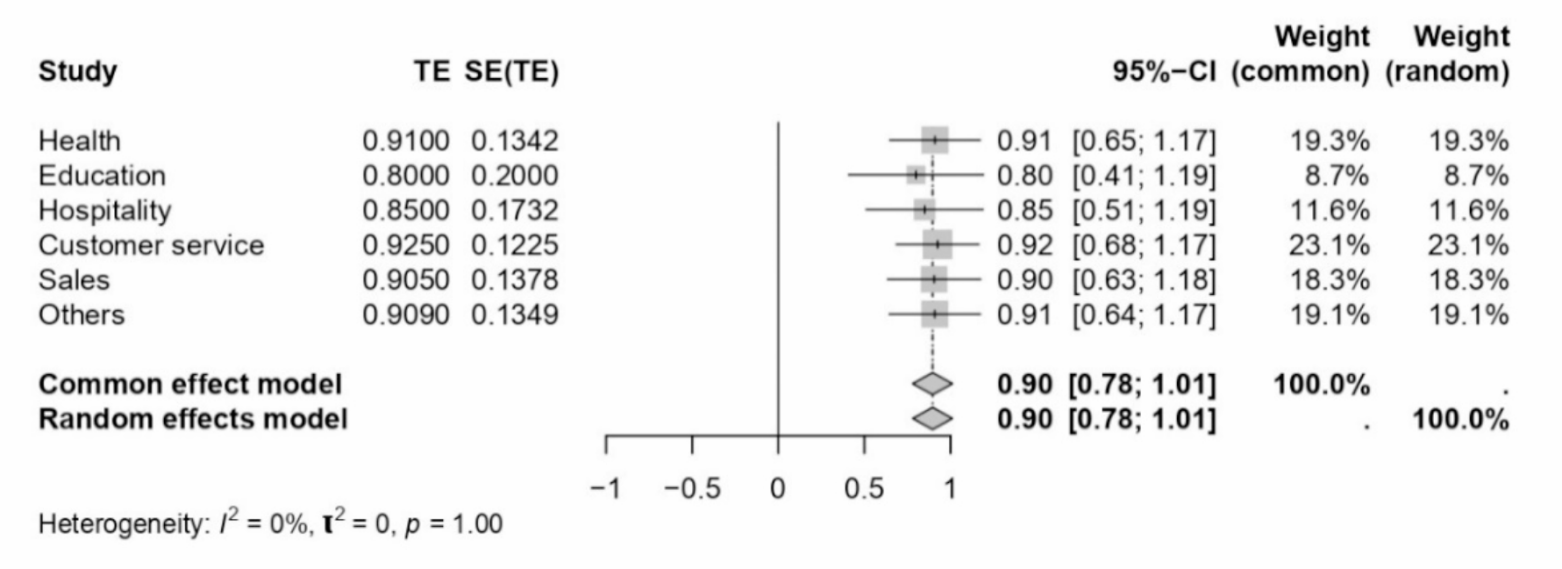
Forest plot concerning sector-related WFC dimension Cronbach’s alphas.

**Fig. 9:**
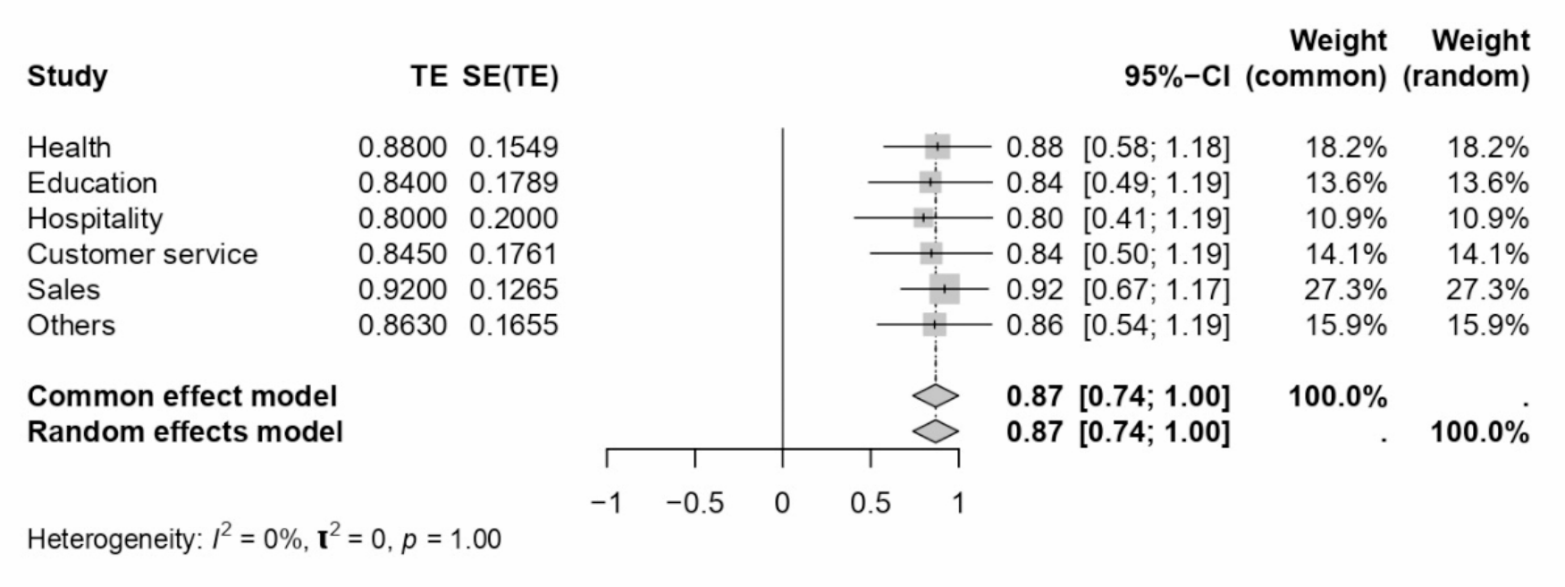
Forest plot concerning sector-related FWC dimension Cronbach’s alphas.

**Fig. 10:**
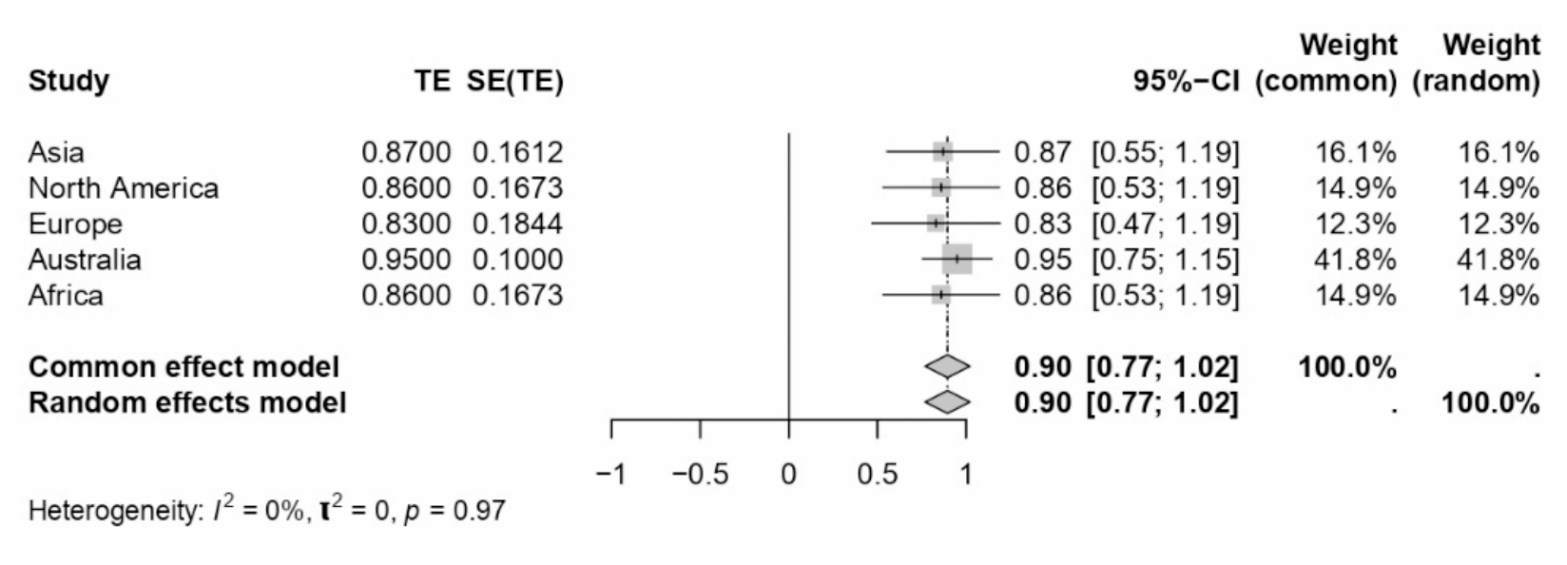
Forest plot concerning location-related FWC dimension Cronbach’s alphas.

A comprehensive meta-analysis examined the internal consistency of Work-Family Conflict (WFC) and Family-Work Conflict (FWC) dimensions during COVID-19 and across various sectors and locations. The study employed Cronbach’s alpha as the estimate for internal consistency across different subgroups defined by sector, location, and periods relative to the COVID-19 pandemic. The results of this analysis are presented in a series of forest plots (see Figs. 7, 8, 9, 4 and 10) and summarised in a subgroup analysis (see Table 2).

In the health sector, WFC and FWC dimensions exhibited Cronbach’s alphas of 0.91 and 0.88, respectively, indicating high internal consistency. The education sector reported lower consistency for WFC (0.80) and FWC (0.84) than the health sector. The hospitality and customer service sectors showed moderate consistency, with alphas ranging from 0.80 to 0.93. Interestingly, the sales sector showed high internal consistency, particularly for FWC (0.92). The “Others” category, encompassing various sectors, demonstrated consistency levels comparable to the health sector, with WFC and FWC alphas of 0.91 and 0.86, respectively.

Asian and European subgroups presented moderate internal consistency for WFC and FWC dimensions. North America showed the highest consistency for WFC (0.92) among the locations analysed. Although limited to single studies, Australia and Africa indicated high internal consistency, particularly Australia, which exhibited a Cronbach’s alpha of 0.95 for both dimensions.

Period Relative to COVID-19, the pre-COVID subgroup analysis revealed lower internal consistency for both WFC and FWC dimensions compared to the COVID and post-COVID periods. During COVID, a single study indicated a Cronbach’s alpha of 0.91 for WFC and 0.88 for FWC. The post-COVID period showed slight improvements in internal consistency, with alphas of 0.90 for WFC and 0.89 for FWC.

## Moderators analysis

For the comparison analysis, two meta-regressions (for gender and age) were conducted in total. See Table 3 for the statistics for all moderator analyses and each meta-regression.

**Table 3:** Comparison meta-regression.

	WFC Estimate (95%CI)									FWC Estimate (95%CI)								
	K	r+	z-value	LL	UL	Tau	Q	I^2^	R^2^(%)	K	r+	z-value	LL	UL	Tau	Q	I^2^	R^2^(%)
Gender (female)	35	61.08	0.00	0.76	0.88	0.06	384.17**	93.73	11.75	35	61.08	1.15	-0.00	0.00	0.07	491.99	93.40	2.05
Age	17	42.25	0.79	-0.00	0.01	0.06	180.54	93.53	0.00	17	42.25	-0.03	-0.01	1.00	0.06	137.88	88.64	0.00

**p < 0.001; K = Number of studies; r + = Gender percentage mean; LL = Lower Limit; UL = Upper limit.

The meta-regression analysis revealed that gender (female percentage) significantly moderated the reliability of the Work-Family Conflict (WFC) scale. Specifically, the analysis, which included 35 studies, found that gender directly impacted the WFC reliability estimates. The high heterogeneity in this analysis, indicated by a Q statistic of 384.17 and an I^2^ of 93.73%, suggests considerable variability across studies, with gender explaining 11.75% of this variance. In contrast, the effect of gender on the Family-Work Conflict (FWC) scale was minimal, not statistically significant, and highly heterogeneous (Q = 491.99, I^2^ = 93.40%). The narrow confidence interval (95% CI: -0.00 to 0.00) and an R^2^ of just 2.05% indicate that gender had little influence on FWC reliability.

As a moderator, age did not significantly affect either scale’s reliability. The analysis across 17 studies showed no significant impact on the WFC scale, with a z-value of 0.79 and a confidence interval (95% CI: -0.00 to 0.01). The heterogeneity remained high (Q = 180.54, I^2^ = 93.53%), and age explained none of the variance (R^2^ = 0.00%). Similarly, for the FWC scale, age showed no significant effect, with a z-value of -0.03 and a confidence interval (95% CI: -0.01 to 1.00). The heterogeneity was slightly lower (Q = 137.88, I^2^ = 88.64%), but again, age contributed nothing to the variance in reliability (R^2^ = 0.00%).

### Sensitivity analyses and robustness of findings

To explore the unusual I^2^ values of 0.0% for both WFC and FWC scales, we conducted several sensitivity analyses to assess potential sources of heterogeneity. Given the diverse contexts of the included studies, we specifically examined the effects of geographical location and sample size on the heterogeneity measures.

First, a geographical location sensitivity analysis revealed that excluding studies from specific regions did not substantially affect the overall heterogeneity. When studies from individual regions were removed, the I^2^ values ranged between 45% and 50%. This suggests that geographical variation did not meaningfully contribute to the heterogeneity in this meta-analysis, indicating consistency in the effect sizes across different regions.

Next, we conducted a sample size sensitivity analysis, which showed that study size was a more important factor in influencing heterogeneity and excluding studies with sample sizes smaller than 200 led to a slight increase in heterogeneity (I^2^ = 49.13%), indicating that smaller studies contributed somewhat less to the variability. When excluding studies with sample sizes smaller than 500, the heterogeneity dropped to 0%, suggesting that the larger studies in the meta-analysis were highly consistent. Conversely, excluding studies with sample sizes larger than 500 increased the heterogeneity to 50.79%, highlighting those smaller studies introduced more variability into the analysis (see Supplementary File 2).

These findings indicate that the initially reported I^2^ values of 0% were likely driven by the inclusion of larger, more consistent studies. In comparison, the smaller studies contributed more variability to the overall meta-analysis.

To further strengthen the robustness of our findings, we performed a leave-one-out sensitivity analysis. This analysis systematically excluded one study at a time to assess the stability of the results. The effect size estimates remained consistent, ranging from 0.8793 to 0.8863, with minimal variation in heterogeneity (I^2^ ranging from 43.57 to 48.44%). Additionally, all effect sizes remained statistically significant (*p* < 0.0001) across the analysis, indicating that no single study disproportionately impacted the overall results. This confirms our meta-analysis’s robustness, as excluding any individual study did not significantly affect the overall effect size or heterogeneity.

In summary, the sensitivity analyses demonstrated that sample size had a greater influence on heterogeneity than geographical location, with smaller studies contributing more variability. The leave-one-out analysis further confirmed the stability and robustness of the meta-analysis results, ensuring that no single study unduly influenced the findings. These analyses collectively support the reliability of the reported effect sizes and heterogeneity estimates.

## Discussion

This meta-analysis provides a comprehensive evaluation of the reliability generalization of the Work-Family Conflict (WFC) and Family-Work Conflict (FWC) scales across diverse geographical locations, occupational sectors, and temporal contexts, including the COVID-19 pandemic. The pooled Cronbach’s alpha values of 0.91 for both WFC and FWC scales emphasise their robust internal consistency, affirming their utility in occupational psychology research. However, a deeper examination of heterogeneity, publication bias, and moderator analyses reveals complex insights that warrant thorough discussion.

One of the striking outcomes of this meta-analysis is the initial report of zero heterogeneity (I^2^ = 0.0%) for both WFC and FWC scales. This finding is particularly unexpected given the substantial diversity among the included studies in terms of geographical regions, cultural contexts, occupational sectors, and the impact of global events like the COVID-19 pandemic. Such uniformity suggests a remarkable consistency in the reliability of these scales across varied settings. However, this primary finding contrasts sharply with the results from sensitivity analyses, which revealed moderate to substantial heterogeneity (I^2^ ranging from 40 to 50%) when studies were stratified by geographical location and sample size.

The discrepancy between the main analysis and sensitivity analyses indicates that the initial low heterogeneity estimate may be driven predominantly by larger studies with consistent reliability estimates, effectively masking variability introduced by smaller studies. When smaller studies (sample sizes < 200) were excluded, heterogeneity increased, highlighting the influence of study size on reliability estimates. Additionally, the leave-one-out sensitivity analysis further demonstrated that excluding individual studies could significantly alter the heterogeneity index, reinforcing the presence of underlying variability not apparent in the overall analysis.

Potential sources of heterogeneity include cultural differences in the perception and reporting of work-family conflicts, variations in organisational policies and support systems, and differences in measurement instruments or administration procedures across studies. For instance, cultural norms regarding work-life balance and family roles may influence how individuals respond to WFC and FWC scales, affecting reliability coefficients. Moreover, organisational factors such as flexibility in work arrangements and support for work-life balance could moderate the consistency of these scales’ reliability across different sectors and regions.

Assessing publication bias is crucial to ensure the validity of meta-analytic findings. In this study, funnel plots for both WFC and FWC scales exhibited slight asymmetry, suggesting a potential mild publication bias where studies reporting higher reliability coefficients may be more likely to be published. However, Egger’s test yielded non-significant results (*p* = 0.93 for WFC and *p* = 0.00 for FWC), indicating no substantial evidence of publication bias. The trim-and-fill method further corroborated these findings by estimating that no studies were missing, and the adjusted pooled Cronbach’s alpha remained unchanged at 0.90.

Despite the non-significant Egger’s test, the slight asymmetry observed in the funnel plots may reflect small-study effects or selective reporting, where smaller studies with lower reliability estimates are underrepresented. This could be due to a publication preference for studies demonstrating high internal consistency, thereby inflating the overall reliability estimates. Nonetheless, the robust pooled estimates and the absence of significant publication bias, as indicated by Egger’s test and the trim-and-fill method, provide confidence in the reliability generalization of the WFC and FWC scales. Future research should continue monitoring publication bias, particularly as more studies emerge from diverse and underrepresented regions.

The moderator analyses revealed that gender significantly influenced the reliability of the WFC scale, with studies featuring a higher percentage of female participants demonstrating different reliability estimates. Specifically, gender accounted for 11.75% of the variance in WFC reliability coefficients, indicating that the scale’s reliability may vary based on gender distribution within samples. This finding aligns with existing literature suggesting that women may experience and report work-family conflicts differently than men, potentially due to differing societal roles and expectations.

In contrast, age did not significantly moderate the reliability of either the WFC or FWC scales, suggesting that the scales maintain consistent reliability across different age groups. However, despite identifying gender as a significant moderator for WFC, a substantial portion of heterogeneity remains unexplained (R^2^ = 2.05% for FWC and negligible for WFC), indicating that other factors contribute to the variability in reliability estimates. Potential unexamined moderators could include organisational culture, economic conditions, technological advancements facilitating remote work, and individual differences such as coping strategies and resilience.

The high heterogeneity uncovered in sensitivity analyses underlines the complexity of factors influencing the reliability of WFC and FWC scales. Future studies should explore a broader range of moderators, including cultural dimensions, sector-specific demands, and the impact of remote and hybrid work arrangements, to understand better the conditions under which these scales perform optimally.

## Implications for practice and research

The consistently high reliability of the WFC and FWC scales across diverse contexts supports their widespread use in occupational psychology research and organisational assessments. Practitioners can confidently employ these scales to measure work-family dynamics, inform interventions, and develop policies to improve employee well-being and organisational performance. However, the identified moderators and sources of heterogeneity highlight the need for contextual considerations when applying these scales. For instance, organisations in regions with distinct cultural norms may need to interpret WFC and FWC scores within local societal expectations and support systems.

From a research perspective, this meta-analysis emphasises the importance of reporting comprehensive reliability metrics, including McDonald’s omega, to complement Cronbach’s alpha and provide a broader assessment of scale reliability. Additionally, future meta-analyses should incorporate a wider array of potential moderators and employ advanced statistical techniques, such as multilevel modelling or meta-regression with multiple moderators, to more effectively parse out the sources of variability in reliability estimates.

### Limitations

Several limitations should be acknowledged. First, excluding non-English studies may introduce language bias, potentially limiting the generalizability of findings to non-English-speaking contexts. Second, the reliance on Cronbach’s alpha as the primary measure of reliability overlooks other psychometric properties that could provide a more comprehensive evaluation of scale performance. Third, the dominance of larger studies in the primary analysis may skew the overall reliability estimates, masking underlying heterogeneity. Finally, despite rigorous quality assessments using QUADAS-2 and COSMIN RB checklists, including studies with varying methodological rigour could influence the meta-analytic results.

### Future directions

Future research should prioritise including non-English studies to enhance the cultural diversity of meta-analytic findings. Additionally, incorporating multiple reliability metrics, such as McDonald’s omega and test-retest reliability, would offer a more robust evaluation of the WFC and FWC scales. Exploring a broader range of moderators, including cultural, organisational, and individual factors, is essential to fully understand the conditions that influence the reliability of these scales. Longitudinal studies examining changes in reliability over time, particularly in response to evolving work practices like remote and hybrid work, would also contribute valuable insights into the dynamic nature of work-family conflicts.

## Conclusion

This meta-analysis reaffirms the high reliability of the WFC and FWC scales across various global contexts, reinforcing their importance as indispensable tools in occupational psychology. While the overall consistency of these scales is commendable, the presence of heterogeneity in sensitivity analyses and the influence of gender as a moderator highlight the need for contextual considerations in their application. Addressing these complexities through comprehensive reporting and exploration of additional moderators will enhance the scales’ utility and ensure their continued relevance in a rapidly changing work environment.

## Electronic Supplementary Material

Below is the link to the electronic supplementary material.

Supplementary Material 1

Supplementary Material 2

Supplementary Material 3

## Data Availability

Data is provided in the supplementary information files.

## Declarations

### Permission to reproduce material from other sources

All material reproduced from other sources has obtained the necessary permissions from copyright holders. References and citations have been meticulously provided throughout the manuscript to acknowledge the original sources, adhering to the journal’s integrity and scholarly rigour standards.
