# Supplementary material for: A meta-analytic evaluation of the reliability of work-family and family-work conflict scales
Source: Sci Rep. 2024 Dec 30;14:31828. doi: 10.1038/s41598-024-83086-z (PMC11686142; doi:10.1038/s41598-024-83086-z)
Supplement: Supplementary file 2 — Supplementary Material 2 [file 41598_2024_83086_MOESM2_ESM.docx]

**Supplemental information**


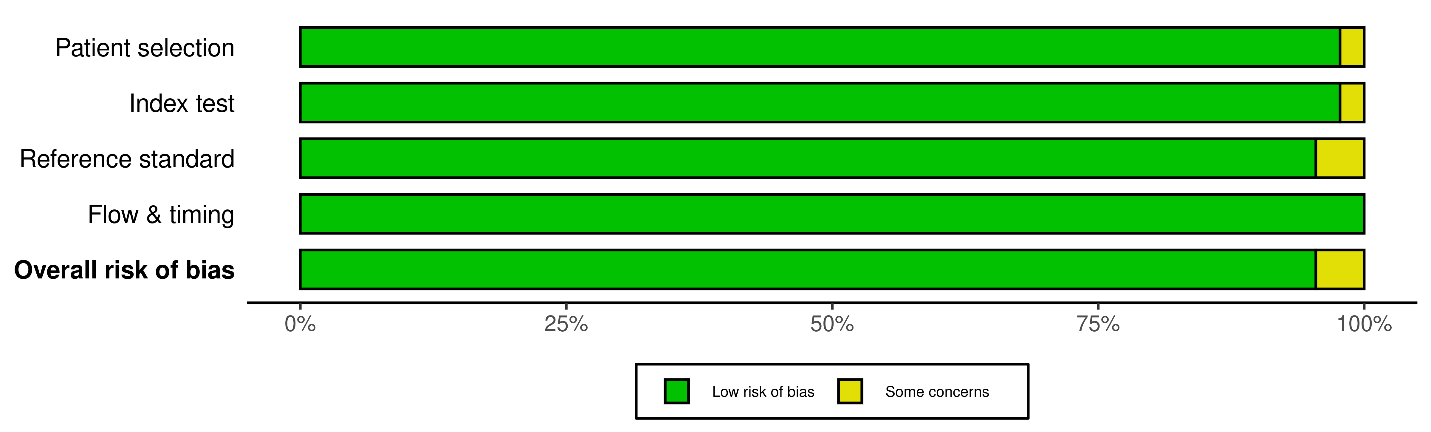


**Fig. 1:** QUADAS-2 Assessments for Included Studies

Table 1. Search terms

| **Search terms** |
| --- |
| ("Reliability " OR "psychometric properties") AND (“[WFC and FWC scales](https://www.emerald.com/insight/content/doi/10.1108/10444060610734163/full/html)” OR "WFC/FWC SCALES") AND ("Cronbach’s alpha" OR "internal consistency" OR "reliability"), TITLE-ABS-KEY(("Reliability generalization" OR "Measurement Properties" OR "Assessment" OR "psychometric properties") AND ("Work-family and family-work scales") AND ( "Measurement Properties" OR "Assessment" OR “Cronbach’s alpha" OR "internal consistency" OR "reliability")), TS=("Measurement Properties" OR "Assessment" OR "Reliability generalization" OR "psychometric properties") AND TS=("Work-family and family-work scales" OR "Measurement Properties" OR "Assessment" OR "[WFC and FWC scales](https://www.emerald.com/insight/content/doi/10.1108/10444060610734163/full/html)") AND TS=("Cronbach’s alpha" OR "internal consistency" OR "reliability")). No language restrictions were imposed, aiming to comprehensively include relevant studies on the reliability of the WFC and FWC scales. |

**Table 2:** Summary of COSMIN Risk of Bias (RB) Checklist Assessments for Included Studies

| **Author (Year)** | **Sample Size** | **Reliability** | **Validity** | **Responsiveness** |
| --- | --- | --- | --- | --- |
| Sharma et al., 2022 | 175 | Adequate | Very Good | Adequate |
| Grant-Vallone & Ensher, 2001 | 118 | Adequate | Adequate | Adequate |
| Boyar et al., 2005 | 432 | Adequate | Doubtful | Adequate |
| Chang & Cheng, 2014 | 463 | Adequate | Adequate | Adequate |
| Rutherford et al., 2014 | 179 | Adequate | Doubtful | Adequate |
| Mary & Ramesh, 2021 | 300 | Very Good | Adequate | Adequate |
| Karatepe & Baddar, 2006 | 20 | Adequate | Adequate | Adequate |
| Karatepe & Kilic, 2007 | 296 | Very Good | Adequate | Adequate |
| Karatepe & Karadas, 2014 | 282 | Very Good | Adequate | Adequate |
| Karatepe, 2009 | 189 | Adequate | Adequate | Adequate |
| Dhingra & Dhingra, 2021 | 206 | Adequate | Adequate | Adequate |
| Cohen et al., 2005 | 414 | Adequate | Adequate | Adequate |
| Cohen, 2009 | 122 | Adequate | Adequate | Adequate |
| Yavas et al., 2007 | 723 | Very Good | Adequate | Adequate |
| Karatepe, 2012 | 110 | Adequate | Adequate | Adequate |
| Karatepe & Karadas, 2016 | 282 | Adequate | Adequate | Adequate |
| Gillet et al., 2022 | 442 | Adequate | Adequate | Adequate |
| Howard et al., 2003 | 119 | Adequate | Adequate | Adequate |
| Rodina et al., 2020 | 181 | Adequate | Adequate | Adequate |
| Komari et al., 2023 | 112 | Adequate | Adequate | Adequate |
| Komlenac et al., 2021 | 274 | Adequate | Adequate | Adequate |
| Su & Jiang, 2023 | 527 | Adequate | Adequate | Adequate |
| Hao et al., 2016 | 931 | Adequate | Adequate | Adequate |
| Fatima et al., 2022 | 206 | Adequate | Adequate | Adequate |
| Turner et al., 2014 | 128 | Adequate | Adequate | Adequate |
| Lim et al., 2021 | 450 | Adequate | Adequate | Adequate |
| Netemeyer et al., 2005 | 320 | Adequate | Adequate | Adequate |
| Netemeyer et al., 2005 | 132 | Adequate | Adequate | Adequate |
| Pennbrant & Daderman, 2021 | 807 | Adequate | Adequate | Adequate |
| Wickramasinghe & Nakandala, 2022 | 380 | Adequate | Adequate | Adequate |
| Rupert et al., 2009 | 487 | Adequate | Adequate | Adequate |
| Li et al., 2019 | 175 | Adequate | Adequate | Adequate |
| Karatepe et al., 2008 | 170 | Adequate | Adequate | Adequate |
| Chang et al., 2014 | 391 | Adequate | Adequate | Adequate |
| Frye & Breaugh, 2004 | 135 | Adequate | Adequate | Adequate |
| Jawahar et al., 2012 | 125 | Adequate | Adequate | Adequate |
| Miao & Wang, 2017 | 320 | Adequate | Adequate | Adequate |
| Karatepe & Uludag, 2008 | 332 | Adequate | Adequate | Adequate |
| Tasdelen-Karckay & Bakalım, 2017 | 356 | Adequate | Adequate | Adequate |
| Karatepe & Azar, 2013 | 141 | Adequate | Adequate | Adequate |
| Weale et al., 2022 | 1416 | Adequate | Adequate | Adequate |
| Simaes et al., 2019 | 310 | Adequate | Adequate | Adequate |
| Pattusamy & Jacob, 2016 | 466 | Adequate | Adequate | Adequate |
| Makola et al., 2015 | 91 | Adequate | Adequate | Adequate |
