# Supplementary material for: A meta-analytic evaluation of the reliability of work-family and family-work conflict scales
Source: Sci Rep. 2024 Dec 30;14:31828. doi: 10.1038/s41598-024-83086-z (PMC11686142; doi:10.1038/s41598-024-83086-z)
Supplement: Supplementary file 3 — Supplementary Material 3 [file 41598_2024_83086_MOESM3_ESM.docx]

**Table for Appendix: Sensitivity Analyses Results**

| **Analysis Condition** | **I² (%)** | **Interpretation** |
| --- | --- | --- |
| **Geographical Location Sensitivity Analysis** | 45-50 | Minimal impact of location on heterogeneity. |
| **Sample Size Sensitivity Analysis** |  |  |
| Excluding studies with sample sizes < 200 | 49.13 | Slight increase in heterogeneity. |
| Excluding studies with sample sizes < 500 | 0 | Larger studies showed consistent results with no heterogeneity. |
| Excluding studies with sample sizes > 500 | 50.79 | Smaller studies contributed more variability. |
| **Leave-One-Out Sensitivity Analysis** | 43.57-48.44 | Robustness confirmed; no single study significantly altered the results. |

**Table: Leave-One-Out Sensitivity Analysis**

| **Excluded Study** | **Estimate** | **SE** | **Z-Value** | **P-Value** | **CI Lower Bound** | **CI Upper Bound** | **I² (%)** | **Tau²** | **Q** | **Q P-Value** |
| --- | --- | --- | --- | --- | --- | --- | --- | --- | --- | --- |
| Study 1 | 0.8828 | 0.0105 | 84.21 | <0.0001 | 0.8623 | 0.9034 | 47.10 | 0.0022 | 79.39 | 0.0004 |
| Study 2 | 0.8810 | 0.0106 | 82.97 | <0.0001 | 0.8601 | 0.9018 | 48.42 | 0.0023 | 81.43 | 0.0003 |
| Study 3 | 0.8798 | 0.0105 | 83.58 | <0.0001 | 0.8592 | 0.9004 | 47.53 | 0.0023 | 80.05 | 0.0004 |
| Study 4 | 0.8863 | 0.0092 | 96.10 | <0.0001 | 0.8682 | 0.9044 | 31.65 | 0.0012 | 61.45 | 0.0267 |
| Study 5 | 0.8793 | 0.0105 | 84.13 | <0.0001 | 0.8588 | 0.8998 | 46.78 | 0.0022 | 78.92 | 0.0005 |
| Study 6 | 0.8823 | 0.0106 | 83.55 | <0.0001 | 0.8616 | 0.9030 | 47.86 | 0.0023 | 80.56 | 0.0003 |
| Study 7 | 0.8840 | 0.0102 | 86.49 | <0.0001 | 0.8639 | 0.9040 | 44.34 | 0.0020 | 75.46 | 0.0012 |
| Study 8 | 0.8817 | 0.0106 | 83.12 | <0.0001 | 0.8609 | 0.9024 | 48.33 | 0.0023 | 81.28 | 0.0003 |
| Study 9 | 0.8800 | 0.0106 | 83.37 | <0.0001 | 0.8593 | 0.9007 | 47.82 | 0.0023 | 80.49 | 0.0003 |
| Study 10 | 0.8826 | 0.0105 | 83.90 | <0.0001 | 0.8620 | 0.9032 | 47.46 | 0.0023 | 79.94 | 0.0004 |
| Study 11 | 0.8820 | 0.0106 | 83.33 | <0.0001 | 0.8613 | 0.9027 | 48.10 | 0.0023 | 80.93 | 0.0003 |
| Study 12 | 0.8835 | 0.0103 | 85.42 | <0.0001 | 0.8632 | 0.9038 | 45.65 | 0.0021 | 77.28 | 0.0007 |
| Study 13 | 0.8812 | 0.0106 | 82.98 | <0.0001 | 0.8604 | 0.9020 | 48.44 | 0.0023 | 81.47 | 0.0003 |
| Study 14 | 0.8812 | 0.0106 | 82.98 | <0.0001 | 0.8604 | 0.9020 | 48.44 | 0.0023 | 81.47 | 0.0003 |
| Study 15 | 0.8796 | 0.0105 | 83.83 | <0.0001 | 0.8590 | 0.9001 | 47.19 | 0.0022 | 79.53 | 0.0004 |
| Study 16 | 0.8800 | 0.0106 | 83.37 | <0.0001 | 0.8593 | 0.9007 | 47.82 | 0.0023 | 80.49 | 0.0003 |
| Study 17 | 0.8793 | 0.0105 | 84.13 | <0.0001 | 0.8588 | 0.8998 | 46.78 | 0.0022 | 78.92 | 0.0005 |
| Study 18 | 0.8805 | 0.0106 | 83.09 | <0.0001 | 0.8597 | 0.9013 | 48.23 | 0.0023 | 81.13 | 0.0003 |
| Study 19 | 0.8806 | 0.0106 | 83.06 | <0.0001 | 0.8598 | 0.9013 | 48.27 | 0.0023 | 81.19 | 0.0003 |
| Study 20 | 0.8795 | 0.0105 | 83.94 | <0.0001 | 0.8589 | 0.9000 | 47.03 | 0.0022 | 79.29 | 0.0004 |
| Study 21 | 0.8812 | 0.0106 | 82.98 | <0.0001 | 0.8604 | 0.9020 | 48.44 | 0.0023 | 81.47 | 0.0003 |
| Study 22 | 0.8811 | 0.0106 | 82.97 | <0.0001 | 0.8603 | 0.9020 | 48.44 | 0.0023 | 81.47 | 0.0003 |
| Study 23 | 0.8807 | 0.0106 | 83.03 | <0.0001 | 0.8599 | 0.9014 | 48.32 | 0.0023 | 81.27 | 0.0003 |
| Study 24 | 0.8797 | 0.0105 | 83.70 | <0.0001 | 0.8591 | 0.9003 | 47.37 | 0.0022 | 79.80 | 0.0004 |
| Study 25 | 0.8798 | 0.0105 | 83.58 | <0.0001 | 0.8592 | 0.9004 | 47.53 | 0.0023 | 80.05 | 0.0004 |
| Study 26 | 0.8809 | 0.0106 | 82.97 | <0.0001 | 0.8601 | 0.9017 | 48.42 | 0.0023 | 81.43 | 0.0003 |
| Study 27 | 0.8805 | 0.0106 | 83.09 | <0.0001 | 0.8597 | 0.9013 | 48.23 | 0.0023 | 81.13 | 0.0003 |
| Study 28 | 0.8798 | 0.0105 | 83.58 | <0.0001 | 0.8592 | 0.9004 | 47.53 | 0.0023 | 80.05 | 0.0004 |
| Study 29 | 0.8796 | 0.0105 | 83.83 | <0.0001 | 0.8590 | 0.9001 | 47.19 | 0.0022 | 79.53 | 0.0004 |
| Study 30 | 0.8810 | 0.0106 | 82.97 | <0.0001 | 0.8601 | 0.9018 | 48.42 | 0.0023 | 81.43 | 0.0003 |
| Study 31 | 0.8810 | 0.0106 | 82.97 | <0.0001 | 0.8601 | 0.9018 | 48.42 | 0.0023 | 81.43 | 0.0003 |
| Study 32 | 0.8798 | 0.0105 | 83.58 | <0.0001 | 0.8592 | 0.9004 | 47.53 | 0.0023 | 80.05 | 0.0004 |
| Study 33 | 0.8833 | 0.0104 | 84.97 | <0.0001 | 0.8629 | 0.9037 | 46.20 | 0.0021 | 78.07 | 0.0006 |
| Study 34 | 0.8821 | 0.0106 | 83.42 | <0.0001 | 0.8614 | 0.9028 | 48.00 | 0.0023 | 80.77 | 0.0003 |
| Study 35 | 0.8805 | 0.0106 | 83.09 | <0.0001 | 0.8597 | 0.9013 | 48.23 | 0.0023 | 81.13 | 0.0003 |
| Study 36 | 0.8793 | 0.0105 | 84.13 | <0.0001 | 0.8588 | 0.8998 | 46.78 | 0.0022 | 78.92 | 0.0005 |
| Study 37 | 0.8819 | 0.0106 | 83.25 | <0.0001 | 0.8611 | 0.9026 | 48.19 | 0.0023 | 81.07 | 0.0003 |
| Study 38 | 0.8835 | 0.0103 | 85.42 | <0.0001 | 0.8632 | 0.9038 | 45.65 | 0.0021 | 77.28 | 0.0007 |
| Study 39 | 0.8807 | 0.0106 | 83.01 | <0.0001 | 0.8599 | 0.9015 | 48.35 | 0.0023 | 81.32 | 0.0003 |
| Study 40 | 0.8835 | 0.0103 | 85.42 | <0.0001 | 0.8632 | 0.9038 | 45.65 | 0.0021 | 77.28 | 0.0007 |
| Study 41 | 0.8796 | 0.0105 | 83.83 | <0.0001 | 0.8590 | 0.9001 | 47.19 | 0.0022 | 79.53 | 0.0004 |
| Study 42 | 0.8842 | 0.0102 | 87.11 | <0.0001 | 0.8643 | 0.9041 | 43.57 | 0.0019 | 74.43 | 0.0015 |
| Study 43 | 0.8800 | 0.0106 | 83.37 | <0.0001 | 0.8593 | 0.9007 | 47.82 | 0.0023 | 80.49 | 0.0003 |
| Study 44 | 0.8805 | 0.0106 | 83.09 | <0.0001 | 0.8597 | 0.9013 | 48.23 | 0.0023 | 81.13 | 0.0003 |
